# Post-operative acute kidney injury in Stanford Type A aortic dissection

**DOI:** 10.1186/1749-8090-8-S1-O13

**Published:** 2013-09-11

**Authors:** GH Chang, T Kofidis

**Affiliations:** 1 Department of Cardiac, Thoracic and Vascular Surgery, National University Health System, Singapore

## Background

Surgical repair is the recommended treatment for acute Stanford Type A aortic dissection. Studies have reported a high incidence of acute kidney injury after surgical procedures of the thoracic aorta in various aortic conditions. This study aims to investigate incidence and risk factors of acute renal failure after surgical repair of Stanford Type A aortic dissection.

## Methods

Over a 10-year period from March 2004 to March 2013, a total of 62 patients underwent open surgical repair of Stanford Type A aortic dissection. We defined acute kidney injury based on the RIFLE criteria. Pre-operative data, intra-operative variables and post-operative outcomes were studied.

## Results

Mean age was 52 ± 12 years. In-hospital peri-operative mortality was 12.9%. Mean aortic cross-clamp time was 124 ± 72 minutes; mean duration of deep hypothermic circulatory arrest (DHCA) was 47 ± 30 minutes. 25.8% of patients developed post-operative acute kidney injury. 6.5% required temporary renal replacement therapy. Pre-operative renal impairment and presence of ischemic heart disease were significant predictors of the need for post-operative hemodialysis. Pre-operative serum creatinine level was a statistical significant risk factor of elevated serum creatinine on the 1st, 2nd and 3rd post-operative day. Duration of DHCA, cardiopulmonary bypass time and body surface area were significant predictors of elevated serum creatinine on the 1st post-operative day. Aortic cross-clamp time correlated significantly wit duration of hemodialysis (p=0.041). Presence of pre-operative renal impairment correlated significantly with the need for hemodialysis (p=0.035). Intra-operative urine output did not correlate significantly with post-operative serum creatinine (p=0.055) or the duration of hemodialysis (p=0.392).

## Conclusions

Identification of the above risk factors allows earlier identification of high risk patients and targeted treatment to minimize the incidence of this complication.

